# Outcome Analysis of the Effects of Helmet Therapy in Infants with Brachycephaly

**DOI:** 10.3390/jcm9041171

**Published:** 2020-04-19

**Authors:** Hyehoon Choi, Seong Hoon Lim, Joon Sung Kim, Bo Young Hong

**Affiliations:** Department of Rehabilitation Medicine, St. Vincent’s Hospital, College of Medicine, The Catholic University of Korea, Seoul 16247, Korea; choihyehoon1021@gmail.com (H.C.); limseonghoon@gmail.com (S.H.L.); joonsungg.kim@gmail.com (J.S.K.)

**Keywords:** brachycephaly, helmet therapy, severity, age, cranial index

## Abstract

Brachycephaly has several potential deleterious effects, including malocclusion, sleep apnea, and abnormal posture. Nevertheless, the research regarding helmet therapy as a treatment strategy for brachycephaly is limited. Herein, we aimed to analyze the factors influencing the effects of helmet therapy in infants with brachycephaly. We retrospectively reviewed the records of 207 infants aged 3–14 months with a cranial index (CI) >90% who received helmet therapy between May 2016 and October 2019 and complied with the treatment protocol well. We used a multiple regression analysis to determine which factors affected the duration of therapy and a Jonckheere–Terpstra test to establish differences in the duration of helmet therapy according to age and severity. We identified brachycephaly severity (*p* < 0.001), asymmetry (*p* < 0.001), and age (*p* < 0.001) as factors affecting the duration of therapy. Helmet therapy might be effective for infants with moderate to severe brachycephaly, assuming good protocol compliance. In addition, younger treatment initiation age and less severe and less asymmetric brachycephaly significantly shorten the treatment duration.

## 1. Introduction

Brachycephaly, defined as the shortened anteroposterior length of the head compared to the width [1], can cause several deleterious effects as well as cosmetic issues. For example, brachycephaly can change the angular orientation of the temporomandibular joint, leading to malocclusion [2,3]. Furthermore, it can cause the anterior displacement of the mandible, resulting in obstructive sleep apnea [4]. Brachycephaly can also change the center of mass of the head, causing an imbalance in the neck flexor and extensor muscles and poor postural stability [3]. One recent report described a case in which brachycephaly caused a reduction in the cranial fossa volume, resulting in the herniation of the hindbrain [5]. Several studies have suggested that positional cranial deformity may be associated with delayed development. Early cognitive and psychomotor developmental delays in craniosynostosis and plagiocephaly infants were reported [6]. A recent study has also shown that brachycephaly is associated with lower cognitive and academic measures in adolescence [7]. The delays were explained with various mechanisms and these associations remain controversial [8,9,10,11]. Collett et al. mentioned the mechanical mechanism in which a cranial deformity leads to the dysmorphology of brain [10], but some have described brain dysmorphology in infants with cranial deformity not being clearly associated with skull shape [8,12]. However, some found the reason for the delayed development of infants with cranial deformity to be environmental factors—restricted movement, sleep position, or the amount of ‘tummy time’ [8,10,13]. A recent study suggested that delayed development could be improved through physiotherapy and orthopedic treatment [9]. Ocular motion abnormalities and otitis media are known to be associated with cranial deformity [14,15]. Rocco et al. reported the higher prevalence of positional plagiocephaly in teenagers, relative to other reports [16]. This result suggests that plagiocephaly might not be corrected spontaneously while growing.

According to the Congress of Neurological Surgeons’ guidelines, published in 2016, helmet therapy should be performed after conservative treatment in children with moderate to severe plagiocephaly that is persistent or detected in a patient at an advanced age [17]. However, some studies have reported that helmet therapy is more effective in younger children and those with less severe cranial deformities [18,19,20]. Moreover, although helmet therapy has been used in children with brachycephaly, most studies have described its use for plagiocephaly. Differing results have been reported in the literature depending on the type of cranial deformity. Teichgraeber et al. stated that helmet therapy was more effective for plagiocephaly than brachycephaly. In this report, 111/111 (100%) children with plagiocephaly achieved normalized head shapes, whereas only 1/33 (3%) children with brachycephaly achieved a normalized head shape when the norm was defined within one standard of age- and sex-adjusted cephalometric variable norms. They attributed this difference to the development of the cranial vault and cranial base. The cranial vault grows in response to the expanding brain mass, but endocranial absorption and ectocranial deposition are responsible for the growth of the cranial base. Compared to plagiocephaly, brachycephaly may be influenced more by cranial base growth [21].

Currently, several aspects of helmet therapy are controversial. First, the instances in which helmet therapy should be applied (e.g., at which age or which level of severity) remain under debate. According to Freudlsperger et al., delayed helmet therapy does not affect the plagiocephaly correction rate in mild cases [22]. However, few previous studies have examined the effects of age and brachycephaly severity on the outcomes of helmet therapy [3,20]. Accordingly, we aimed to investigate the factors that influence the effects of helmet therapy with proper treatment compliance in children with moderate to severe brachycephaly. Additionally, we investigated the factors that could potentially affect the duration of brachycephaly treatment.

## 2. Methods

We retrospectively reviewed the records of infants with a cranial index (i.e., cephalic index; CI) > 90% who received helmet therapy and completed follow-up between May 2016 and October 2019. The CI was calculated by dividing the biparietal diameter of the head by its occipitofrontal diameter and multiplying this ratio by 100 [23]. To ensure that our study included only patients who complied well with treatment, those who did not wear a helmet for more than 2 months before they attained a CI of less than 90% or did not complete the follow-up period were excluded from the analysis. The age at treatment initiation was recorded as the nearest whole month postpartum after correcting for prematurity (if applicable). Patients were then assigned to groups based on age and brachycephaly severity (Table 1). The children were divided into five groups according to age. The age standard was set in consideration of other papers and the slope of head circumference [3,20,24,25]. The slope of the head circumference growth is very steep in the early infant period. However, the slope becomes smooth from around 10 months, and remains similar up to 24 months [26]. We divided the brachycephaly severity groups by referring to previous studies and with the consideration of the patient numbers in each group [3,21,27]. The study protocol was approved by the Institutional Review Board (IRB Number: VC20DASI00**) of the Catholic University of Korea, St. Vincent’s Hospital, which waived the requirement for patient consent.

### 2.1. Helmet Therapy

**Figure 1:**
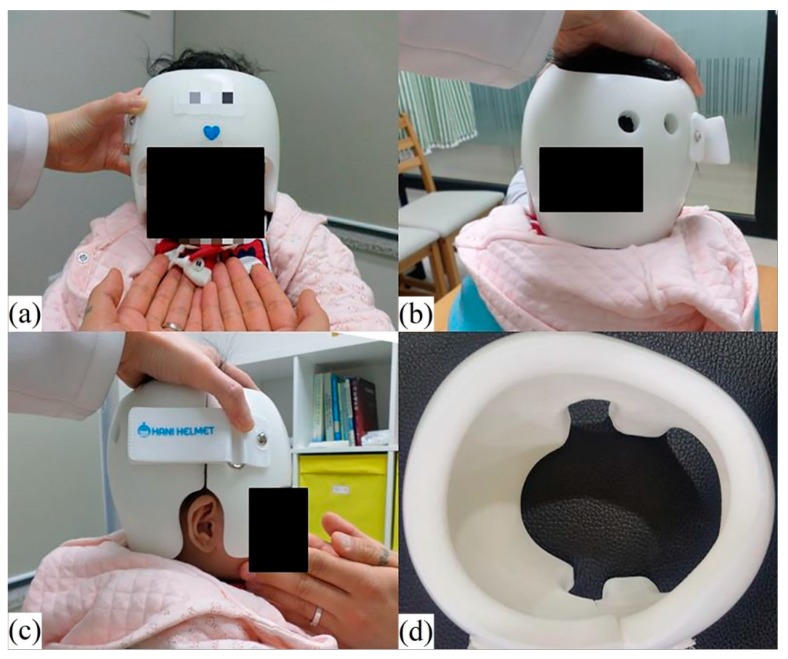
Clinical photograph of an infant wearing a helmet. A helmet, composed of polypropylene (outside) and polyethylene foam (inside), was adjusted to the head of each patient. (**a**) Front view (**b**) Posterior view (**c**) Side view (**d**) Inside view of the helmet.

**Figure 2:**
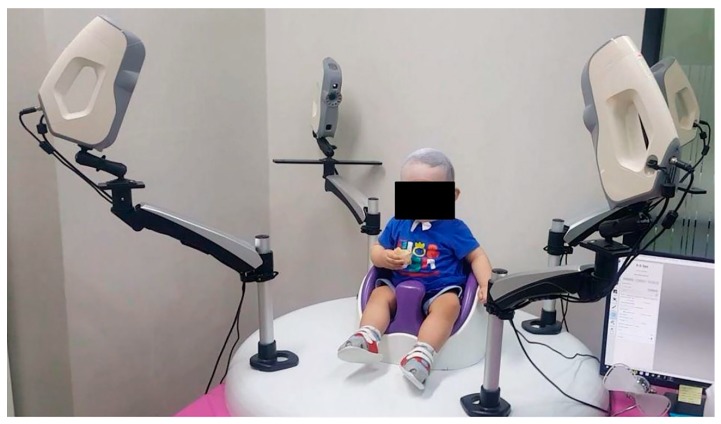
Clinical photograph of the process of scanning for skull deformities. The frequent movement of the infants increased the difficulty of obtaining accurate images. The simultaneous use of four three-dimensional scanners enabled the acquisition of precise images over an imaging duration of only 0.5–0.8 s.

**Figure 3:**
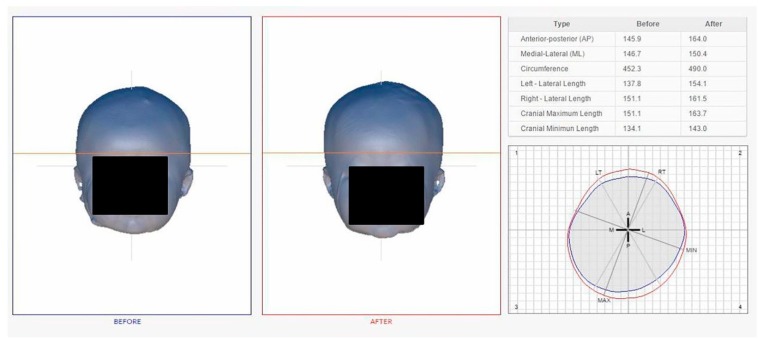
Software-assisted comparison of the head shape values before and after treatment. Cephalometric variables were measured by dividing by head into ten segments from the top of the head to the chin. The measurements were conducted monthly, and the helmet was adjusted accordingly.

Patients in this study were treated using a helmet (Han Health Care Inc., Seoul, Korea) comprised of an external polypropylene shell and internal polyethylene foam layer. The helmet was customized to the head size of each patient (Figure 1). The severity of skull deformation was assessed using four Eva Lite 3D Scanners (Artec, Luxembourg, Luxembourg) to compensate for the frequent movement of infants (Figure 2). The corresponding software package -Artec Studio (Artec, Luxembourg, Luxembourg) and a HANI viewer (Han Health Care Inc., Seoul, Korea) were used to make several measurements of the head, including the anterior–posterior and medial–lateral dimensions, circumference, diagonal diameter, and cranial maximum and minimum lengths (Figure 3). The software measured the cephalometric values by dividing the head into ten segments, from the crown of the head to the chin. The left and right diagonal diameters were measured at an angle of 30° from the center of the nose and the outer edge of the eyebrow. The cranial vault asymmetry index (CVAI, %) was calculated by dividing the difference between longer and shorter diagonal diameter by the shorter diagonal diameter and multiplying this ratio by 100 [23]. The measurements were conducted monthly and the helmet was adjusted following each measurement by the manufacturer.

### 2.2. Statistical Analysis

All statistical analyses were performed using SPSS version 21 (IBM Corp., Armonk, NY, USA). The Shapiro–Wilk and Kolmogorov–Smirnov tests were used to determine the normality of the data. A paired t-test was used to identify differences between the initial and final CIs. A multiple regression analysis was conducted to investigate factors that could potentially affect the treatment duration, including sex, prematurity, delivery method, birth status (single vs. multiple), age at treatment initiation, initial CI, and initial CVAI. The first model analyzed all the factors, and the second model included only the variables that were determined to be statistically significant by the first model. We applied the Jonckheere–Terpstra test to investigate trends in the treatment duration based on age and severity. All tests were two-tailed, and a *p*-value of <0.05 after the Bonferroni correction was considered statistically significant.

## 3. Results

**Figure 4:**
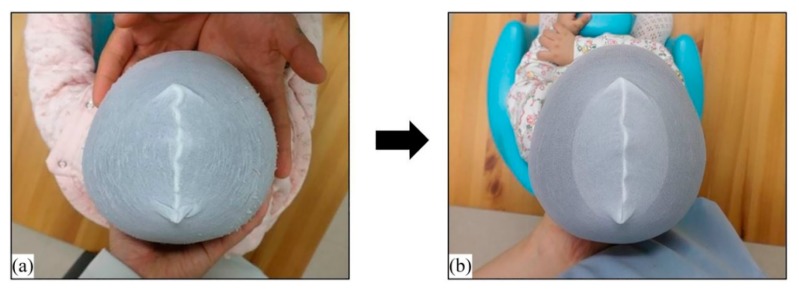
A 4-month-old male infant with brachycephaly (**a**). The patient’s CI was within the normal range after 4 months of orthotic helmet therapy (**b**).

The records of 845 infants whose helmets were made from May 2016 to October 2019 were reviewed, and 725 infants had brachycephaly with a CI > 90% at initial measurement. Among these, 273 infants were not contacted in the middle of therapy, 178 infants did not wear the helmet for 2 or more months due to parental reasons or the infant’s refusal (without any health issue), 55 infants stopped wearing the helmet due to health problems, such as hospital admission or surgery, and 12 infants moved to another area or emigrated. Finally, 207 infants with a CI > 90% who complied with treatment protocol were included in the study. Table 2 presents the demographic data of the study participants. The study population included 132 boys (64%) and 75 girls (36%). Ninety-nine infants (52%) were delivered vaginally and 108 infants were born by cesarean section (48%). Sixteen infants (8%) were born prematurely, whereas 31 infants (15%) were twins. Of the 207 infants, 204 (98.5%) exhibited a decrease in CI by < 90% after therapy (Figure 4). In a paired t-test, patients in all age and severity groups exhibited significant improvements relative to the initial CI (mean initial CI: 95.55 ± 3.38%, final CI: 88.87 ± 1.07%), so that the final CI differed significantly from the initial CI (Table 3). In a multiple regression analysis, the initial CI (*p* < 0.001, β = 0.451), initial CVAI (*p* < 0.001, β = 0.185), and age at treatment initiation (*p* < 0.001, β = 0.389) were identified as factors significantly associated with the treatment duration (Table 4). Furthermore, the Jonckheere–Terpstra test indicated prolonged treatment duration in patients with more severe brachycephaly, regardless of age, as well as in older patients, regardless of severity (Table 5).

The first model included all variables; the second model included only the variables that were determined to be statistically significant by the first model.

## 4. Discussion

Our results demonstrate that helmet therapy led to improved CIs in infants aged 3–14 months with moderate to severe brachycephaly who adhered well to the treatment protocol. Moreover, we identified younger treatment initiation age and less severe and asymmetric brachycephaly as factors that shortened the treatment duration in cases with similar final CIs. We assigned participants to subgroups based on age and brachycephaly severity and explored whether the efficacy of helmet therapy differed according to these variables. In a study by Kelly et al., 81.4% of patients with brachycephaly achieved a final CI <90% after helmet therapy [3]. In contrast, our study showed a success rate of 98.5%. We note that although the patients in these two studies had similar ages at treatment initiation and initial CIs (Kelly et al. vs. our study: age at initiation, 5.8 ± 1.5 vs. 5.61 ± 2.67 months; initial CI, 95.0 ± 3.2% vs. 95.55 ± 3.37%), the average duration of treatment was longer in our study (13.5 ± 5.7 weeks vs. 5.02 ± 2.18 months). Moreover, we excluded patients who did not adhere to the therapy guidelines (i.e., not wearing the helmet for more than 2 months before they attained a CI of less than 90%, incompletion of follow-up), whereas Kelly et al. did not exclude such patients. Therefore, the higher rate of success in our study may be attributable to the higher compliance and longer duration of therapy. 

We divided the participants according to age and severity based on the number of patients and methods used in previous studies. Cevik at el. showed that starting helmet therapy before the age of 6 months improved the outcome [18]. The Congress of Neurological Surgeons’ guidelines recommend starting helmet therapy at a later age (usually over 8 months of age) [17]. We set the highest cutoff age at 10 months, based on the growth of head circumference and other papers. The study by Kelly et al. was used as a reference to classify the participants based on the severity of brachycephaly [3]. They classified 88 < CI ≤90 as mild, 90 < CI ≤ 93 as moderate, and CI > 93 as severe brachycephaly. We also created a very severe group (CI > 96), considering the number of patients. Further studies are needed to define appropriate criteria based on age and the severity of brachycephaly. In our sample, the oldest age group cutoff was 10 months old and the highest brachycephaly severity group cutoff was a CI of > 96%. Because this study included only five children over 12 months old, further study is needed to investigate the effectiveness of helmet treatment in children whose first-year head growth spurt has passed and whose anterior fontanelle has nearly closed. 

The proportion of boys was almost twice that of girls in this study. Previous studies on patients with plagiocephaly have also reported a male predominance [28,29]. Some studies have attributed this observation to the larger and less flexible heads of male infants [28], which might explain the male predominance observed in this study. Half the participants were vaginally delivered, and the other half delivered by cesarean section. Vacuum/forceps delivery is known to be a risk factor for cranial deformity, especially plagiocephaly [28,30], but the records used in this study did not include data regarding vacuum/forceps delivery. Furthermore, the logistic regression analysis revealed that delivery method, prematurity, and multiple births did not influence the duration of helmet therapy. Recently, Hinken et al. conducted an analysis of covariance to determine which factors affect the final CI in children with brachycephaly and plagiocephaly, and identified how the treatment duration, age at treatment initiation, baseline CI, and compliance affect the final CI [20]. However, Hinken et al. did not include the initial CVAI as an independent variable. In our study, we assessed the effects of the initial CI, age at treatment initiation, and initial CVAI on therapy duration. With every one-month-old increase in the age at treatment initiation, we observed an increase of 0.391 months in treatment duration. Also, with every 1% increase in the initial CI (%) and initial CVAI (%), an increase of 0.453, and 0.187 months in treatment duration was observed, respectively. The effects of the initial CVAI on treatment duration can be explained by the requirement for anteroposterior and/or mediolateral redirection. Although mediolateral redirection of the head is not necessary in infants with symmetric brachycephaly, asymmetric brachycephaly requires a redirection in both the anteroposterior and mediolateral directions. 

Although our study highlights some important aspects of brachycephaly and helmet therapy, it has several limitations. Firstly, this was a retrospective rather than a randomized controlled study. Some different therapies have been used to treat cranial deformity—repositioning therapy, physiotherapy, osteopathic manipulation, and surgery, as well as helmet therapy [8]. Graham et al. reported that cranial orthotic therapy was more effective than repositioning therapy [27]. Our study further supports the effectiveness of helmet therapy, assuming good protocol compliance. Recent studies showed no statistically significant difference between helmet therapy and physiotherapy, but the authors advised the use of a combination of both techniques, depending on various factors [31]. However, there was no control group in this study to confirm that the improvement was the result of helmet therapy alone, without any counter-positioning or physiotherapy. Therefore, a well-designed randomized controlled study comparing helmet therapy to other therapies is needed to confirm the effects of helmet therapy. Secondly, the final CI should not be used to evaluate the outcome, because most patients in this study achieved a final CI < 90%, at which point treatment ceased, and the final CIs of most patients were similar, at approximately 90% (88.87 ± 1.07). We note that Graham et al. found no significant association between age and therapy duration in patients with mild (CVAI < 6.25) plagiocephaly [24]. Although brachycephaly has been defined as a CI > 81% [1], our study only included cases of moderate to severe brachycephaly, classified as a CI > 90%; thus, patients with mild brachycephaly, classified as a CI of 81%–90%, were not included. Further studies are needed to establish whether similar trends in efficacy would be observed in patients with mild brachycephaly. Thirdly, selection bias may have affected the effects of helmet therapy. We only included patients who had moderate to severe brachycephaly (CI > 90%) and who complied well with the treatment protocol in a single center. One of the most critical factors in determining the effects of helmet therapy is compliance; however, the degree of compliance varies among children and the variation is considerable. This study investigated factors affecting the results under the assumption that all the participants had proper compliance. The rate of dropout was quite high in the database, as it was over 50%. There are some presumable reasons for this low adherence. First of all, the initial treatment effect might have influenced the adherence to the therapy. Secondly, it is likely that the medical complications of brachycephaly were not serious enough to endure the discomfort and annoyance of the treatment. Deformational plagiocephaly is considered to be a risk for developmental delays [32,33]; however, the harmful effects of brachycephaly are controversial [8,9]. Recently, deformational plagiocephaly has been regarded as a consequence of early developmental delays, rather than the cause of delayed development [8,34]. As the patient records were written by the helmet manufacturer, not at the hospital, they did not consistently include medical symptoms such as developmental, sleep, postural problems, or other medical symptoms. Therefore, we were not able to deduce the developmental outcome or other medical aspects that are known to be related to brachycephaly. However, children with serious medical problems were likely excluded because the manufacturer was required to obtain a doctor’s approval that helmet treatment was appropriate. Therefore, we speculate that many parents might have chosen helmet treatment for cosmetic purposes rather than medical situations. This might be one of the reasons for the high dropout rate. Furthermore, it might have been inconvenient to visit the center and adjust the helmet every month. Thirdly, in the same context, it is possible that the parents voluntarily stopped visiting the center when the deformation was corrected to a satisfiable extent. Finally, one of the most common sources of discomfort due to wearing a helmet is sweating, which could also have been a factor for dropouts during warm seasons.

Despite these limitations, this was the first study to analyze the effects of helmet therapy in infants with brachycephaly, according to both age and severity. Our results demonstrate that helmet therapy is an appropriate treatment strategy for children younger than 14 months old and those with moderate to severe brachycephaly (CI > 90%). Moreover, we analyzed the factors that affect treatment duration, thus providing useful information to caregivers, medical staffs and helmet makers regarding helmet therapy in infants with brachycephaly.

## 5. Conclusions

Our study reveals that helmet therapy might be effective in children aged 3–14 months with moderate to severe brachycephaly, assuming good treatment compliance. The results also highlight the association of the severity of brachycephaly, the severity of asymmetry, and age at treatment initiation with the duration of helmet therapy. Younger children with less severe cases required significantly shorter treatment durations for successful outcomes. Further studies intended to establish comprehensible guidelines for helmet therapy in patients with brachycephaly are needed.

## Figures and Tables

**Table 1:** Classification of subjects according to brachycephaly severity and age at treatment initiation (*n* = 207).

**Classification According to Brachycephaly Severity**							
Cranial index (%)	90–93		93–96			>96	
Classification	Moderate		Severe			Very severe	
Number of subjects	51		62			91	
**Classification According to Age at Treatment Initiation**							
Age at treatment initiation (months)	<4	4–5		6–7	8–9		≥10
Classification	Very early	Early		Mid	Late		Very late
Number of subjects	39	73		51	26		14

**Table 2:** Demographics and clinical features of patients.

	**Mean ± S** **tandard Deviation (Range)**
Age at initiation (months)	5.61 ± 2.67 (3–14)
Treatment duration (months)	5.02 ± 2.18 (1–13)
Initial CVAI (%)	8.11 ± 3.25 (1.00–16.27)
Initial CI (%)	95.55 ± 3.37 (90.10–106.10)
	**Number of Children** **(%)**
**Sex**	
Male	132 (64%)
Female	75 (36%)
**Method of delivery**	
Vaginal delivery birth	99 (52%)
C-section birth	108 (48%)
**Gestational age**	
Mature (born at ≥ 37 weeks)	191 (92%)
Premature (born at < 37 weeks)	16 (8%)
**Birth number**	
Single	176 (85%)
Twin	31 (15%)

Cranial index (CI), cranial vault asymmetry index (CVAI).

**Table 3:** Effects of helmet therapy according to age at treatment initiation and severity of brachycephaly (paired t-test).

	Number	Initial CI, %	Final CI, %	*p*-Value
		(Mean ± SD)	(Mean ± SD)	
Total	207	95.55 ± 3.38	88.87 ± 1.07	<0.001 *
Age (months)				
Very early (<4)	41	95.40 ± 3.23	88.90 ± 1.07	<0.001 *
Early (4–5)	73	96.33 ± 3.15	89.00 ± 0.95	<0.001 *
Mid (6–7)	52	95.82 ± 3.60	88.80 ± 1.04	<0.001 *
Late (8–9)	27	94.37 ± 3.56	88.72 ± 1.27	<0.001 *
Very late (≥10)	14	93.18 ± 2.26	88.71 ± 1.38	<0.001 *
Severity (CI %)				
Moderate (90 to < 93)	51	91.41 ± 0.84	87.69 ± 1.21	<0.001 *
Severe (93 to < 96)	62	94.34 ± 0.86	89.12 ± 0.74	<0.001 *
Very severe (>96)	94	98.55 ± 2.18	89.35 ± 0.60	<0.001 *

Standard deviation (SD), cranial index (CI), * *p* < 0.05.

**Table 4:** Multiple linear regression analysis of factors potentially associated with treatment duration in patients with a CI < 90% (*N* = 204).

**1st Model**						
Variables	β Coefficient	95% Confidence Interval for β Coefficient			Standard	*p*-Value
		Lower	Upper		Error	
Age at	0.389	0.301		0.475	0.044	<0.001 *
treatment initiation						
Initial CI	0.451	0.384		0.52	0.034	<0.001 *
Initial CVAI	0.185	0.115		0.254	0.035	<0.001 *
Sex	−0.027	−0.442		0.385	0.21	0.898
Prematurity	−0.088	−0.857		0.689	0.392	0.822
Method of delivery	−0.006	−0.424		0.403	0.21	0.978
Multiple births	−0.041	−0.633		0.545	0.299	0.89
Model characteristics	*R* = 0.725, *R*^2^ = 0.525, adjusted *R*^2^ = 0.508,					
	*F* = 30.983, *p* < 0.001					
**2nd Model**						
Variables	β Coefficient	95% Confidence Interval for β Coefficient			Standard	*p*-Value
		Lower	Upper		Error	
Age at	0.391	0.306		0.475	0.458	<0.001 *
treatment initiation						
Initial CI	0.453	0.387		0.519	0.763	<0.001 *
Initial CVAI	0.187	0.119		0.254	0.302	<0.001 *
Model characteristics	*R* = 0.725, *R*^2^ = 0.525, adjusted *R*^2^ = 0.518,					
	*F* = 73.710, *p* < 0.001					

Cranial index (CI), cranial vault asymmetry index (CVAI), * *p* < 0.05.

**Table 5:** Therapy duration according to the age and severity (Jonckheere–Terpstra test).

**Mean Treatment Duration (Months) (Mean ± SD)**							
		Age at helmet therapy initiation					
		Very early	Early	Mid	Late	Very late	P-trend’ for severity
Initial severity (CI %)	Moderate (90 to < 93)	1.47 ± 0.74	2.21 ± 1.01	2.65 ± 1.29	2.16 ± 1.37	3.49 ± 2.53	0.036 *
	Severe (93 to < 96)	3.02 ± 1.09	3.84 ± 1.09	4.77 ± 1.50	4.71 ± 1.94	6.81 ± 3.55	<0.001 *
	Very severe (>96)	3.81 ± 1.11	4.28 ± 1.34	6.22 ± 1.54	6.13 ± 2.18	7.6	<0.001 *
	P-trend for age	<0.001 *	<0.001 *	<0.001 *	<0.001 *	0.006 *	

Cranial index (CI), standard deviation (SD), * *p* < 0.05.
